# Acute stress induces changes in epigenome-wide DNA methylation

**DOI:** 10.1038/s41386-025-02289-8

**Published:** 2025-12-12

**Authors:** Lea Zillich, Nathaly S. Czernin, Oscar Crespo Salvador, Elisabeth M. Hummel, Paul Pauli, Andreas Reif, Jürgen Deckert, Katharina Domschke, Miriam A. Schiele

**Affiliations:** 1 Department of Psychiatry and Psychotherapy, Medical Center—University of Freiburg, Faculty of Medicine, University of Freiburg, Freiburg, Germany; 2 Department of Psychology I (Biological Psychology, Clinical Psychology and Psychotherapy), University of Würzburg, Würzburg, Germany; 3 Department of Psychiatry, Psychosomatic Medicine and Psychotherapy, University Hospital Frankfurt – Goethe University, Frankfurt, Germany; 4 Department of Psychiatry, Psychosomatics and Psychotherapy, Center of Mental Health, University Hopsital Würzburg, University of Würzburg, Würzburg, Germany; 5 Institute for Clinical Epidemiology and Biometry, University of Würzburg, Würzburg, Germany; 6 German Center for Mental Health (DZPG), partner site Berlin/Potsdam, Berlin/Potsdam, Germany

**Keywords:** DNA methylation, Psychology, Stress and resilience

## Abstract

Stress plays a significant role in the development of mental and somatic disorders by dysregulating the hypothalamic-pituitary-adrenal (HPA) axis. Epigenetic mechanisms, particularly DNA methylation (DNAm), are assumed to mediate this relationship, with increasing evidence linking stress experience to DNAm changes, though the longitudinal effects of acute stress remain unclear. Here, 122 healthy individuals underwent the Maastricht Acute Stress Test (MAST). Salivary samples for cortisol measurements were taken at seven time points from before to 45 min after stress induction, and blood was drawn for DNAm analyses before and 45 min after. Cortisol reactivity was predicted by baseline DNAm using robust linear models, and a mixed linear model was performed to investigate DNAm changes over time. Downstream analyses included identifying differentially methylated regions (DMRs) and overrepresented Gene Ontology (GO) Terms. A total of 120 CpG sites and four DMRs were associated with cortisol reactivity, with chromatin modifiers among the top findings. Longitudinal epigenome-wide changes were observed at 32 CpG sites and four DMRs. Overrepresented GO terms were related to learning, cognition, and synaptic processes. Two thyroid-related genes, *TTR* and *TSHR*, were observed among the top hits. These findings highlight the association between acute stress and DNAm, suggesting DNAm levels to be related to cortisol reactivity and acute stress to influence DNA methylation patterns dynamically. Key genes involved in thyroid function and transcriptional regulation were implicated in the stress response. Further research with larger samples and multi-omics approaches is needed to confirm these findings, assess their long-term stability, and explore their functional relevance.

## Introduction

Stress is a major driver of psychopathology and has been shown to play a significant role in the development and exacerbation of various mental and somatic disorders [1]. After exposure to acute stress, the hypothalamic-pituitary-adrenal (HPA) axis is activated, releasing stress hormones such as cortisol. While this is an adaptive response to acute stress, after prolonged stress exposure, chronic HPA axis dysregulation is observed, which has been associated with mental disorders [2, 3]. For instance, a subgroup of patients with major depressive disorder appears to be characterized by impaired HPA axis functioning and thus might benefit from pharmacological treatments targeting the HPA axis [4]. One of the mediating biological mechanisms that could explain the relationship between stress, HPA axis dysregulation, and the development of stress-related mental disorders is epigenetics [5, 6].

Epigenetics includes mechanisms that impact gene regulation without changing the DNA sequence itself. An often-studied epigenetic mechanism is DNA methylation, which refers to the addition of a methyl group in the 5‘ position of a cytosine base, most often occurring in cytosine-guanine dinucleotides (CpGs) [7]. While DNA methylation can be stable in the form of an epigenetic memory, overall, it reflects a dynamic process that external factors, such as environmental exposure and stress experience, can influence [8]. Therefore, DNA methylation is ideally suited to be investigated longitudinally in experimental procedures.

In earlier studies, the role of DNA methylation in acute stress was investigated using candidate gene approaches, for instance, targeting genes in the HPA axis, such as the glucocorticoid receptor (*NR3C1*) [9] and the FK506 binding protein 51 (*FKBP5*) genes [10], and other stress-related genes such as the brain-derived neurotrophic factor (*BDNF*) and the oxytocin receptor (*OXTR*) [11, 12]. While these studies have produced preliminary evidence for the association between acute stress reactivity and DNA methylation, candidate gene studies are prone to false positive associations [13], underlining the need for epigenome-wide investigations of DNA methylation signatures in response to acute stress.

On an epigenome-wide level, there is increasing evidence that stressful life events can impact DNA methylation, even in utero [14]. While DNA methylation changes have been associated with stress-related mental disorders [15], including affective disorders [16], anxiety disorders [17], and posttraumatic stress disorder [18, 19], it is still largely unknown how DNA methylation changes under acute stress. A first epigenome-wide association study of cortisol reactivity in response to acute laboratory stress induction in individuals with a history of childhood trauma revealed no significant associations after multiple testing correction [20]. However, one of the strongest signals, a CpG site in the KIT Ligand (*KITLG*) gene exhibiting a strong association with cortisol reactivity, was replicated in independent samples [20]. While this study provides an important basis, the longitudinal dynamics of DNA methylation in response to acute stress remain unclear. Therefore, the aim of the present study was (i) to examine whether DNA methylation levels predict cortisol reactivity under acute stress and (ii) to investigate longitudinal DNA methylation changes in response to acute stress in healthy volunteers.

## Materials and methods

### Sample

A total of 122 healthy participants (mean age 28.06 years, SD = 7.5 years) were recruited from the CRC-TRR58 subproject Z02 in Würzburg, Germany (cf. [21–23]). The inclusion and exclusion criteria were consistent with those applied to the broader Z02 sample, as detailed in previous publications [21–23]. In brief, exclusion criteria were a current or lifetime DSM-IV axis I diagnosis, severe somatic or neurological conditions, non-European ancestry (self-reported up to the third generation), consumption of excessive amounts of caffeine, alcohol, or nicotine (assessed via self-report), the use of illicit drugs, intake of psychoactive medication, pregnancy, and cold intolerance. All participants gave written informed consent and received a compensation of 15€. The study was approved by the ethics committee of the medical faculty at the University of Würzburg, Germany (vote no. 133/14), and was conducted in line with the Declaration of Helsinki.

### Maastricht acute stress test and biomaterial collection

The Maastricht Acute Stress Test (MAST) is a standardized stress paradigm to induce acute stress in a laboratory setting [23, 24]. In brief, participants underwent ten minutes of acute stress induction after five minutes of stress anticipation. Here, five hand immersion trials were alternated with four mental arithmetic tasks. In the hand immersion trials, participants had to submerge their hands into ice-cold water (2 °C). In the arithmetic trials, participants counted backward from 2043 in steps of 17. As a social stressor, all participants were videotaped during the stress induction. All sessions were conducted in the afternoon between 1:00 pm and 4:30 pm to control for circadian variability in cortisol levels as well as DNA methylation.

Salivary cortisol samples were collected at seven time points: before stress induction (T0), directly after the acute stress phase (T1), and 5 (T2), 10 (T3), 20 (T4), 30 (T5), and 45 min (T6) following stress offset. All salivary samples were collected using Salivette® Cortisol swabs (Sarstedt, Nümbrecht, Germany).

**Fig. 1:**
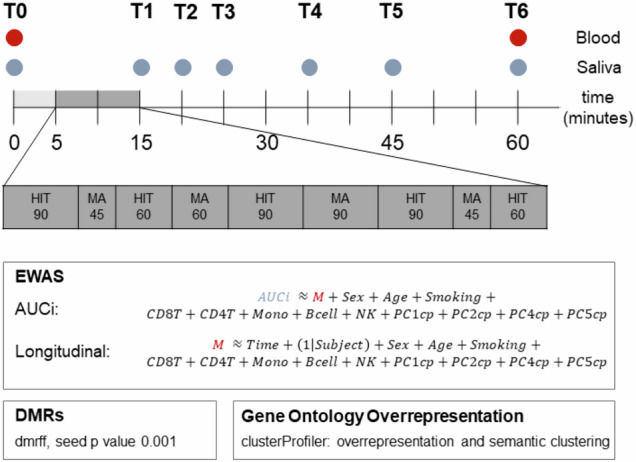
Maastricht acute stress test (MAST) protocol and bioinformatics workflow. HIT hand immersion trials, MA mental arithmetric trials, AUCi area under the curve with respect to cortisol increase, M methylation M value, CD8T CD8 positive T cell estimate, CD4T CD4 positive T cell estimate, Mono monocyte estimate, Bcell B cell estimate, NK natural killer cell estimate, PC1cp-PC5cp principal component 1–5 of the internal control probes, DMR differentially methylated region.

Blood was drawn at T0, i.e., before stress induction, and at T6, i.e., 45 min after the MAST. The experimental workflow is depicted in Fig. 1.

### Cortisol analysis

After completion of the experimental procedure, saliva samples were refrigerated at 4 °C and processed within seven days of collection. Then, samples were centrifuged for ten minutes at 3500 rpm and stored at –80 °C until further processing. After sample collection was complete, all samples were thawed and centrifuged for three minutes at 3000 rpm. Commercially available chemiluminescence-immunoassays (CLIA; IBL, Hamburg, Germany) were used to measure salivary-free cortisol concentrations at the Department of Biopsychology, Technical University of Dresden, Germany. Cortisol levels were log-transformed, and cortisol reactivity was determined for each participant as the area under the curve with respect to increase (AUCi [25]) from T0 to T6. Data from two participants were excluded because cortisol levels were missing at single time points.

### DNA methylation and preprocessing

DNA was isolated from frozen whole blood using the FlexiGene DNA Kit (QIAGEN, Hilden, Germany) and stored at –80 °C until further processing.

DNA methylation levels at approximately 865,000 sites were determined using the Illumina Infinium EPIC BeadChip v1.b5 (Illumina, San Diego, CA, USA) following bisulfite conversion. For each individual, DNA samples from both timepoints were analyzed on the same chip to avoid batch effects in the longitudinal analysis. Bisulfite conversion, hybridization, and processing were performed according to the manufacturer’s instructions at Life & Brain GmbH, Bonn, Germany.

Raw data was stored in .idat files. A customized version of the CPACOR pipeline [26] was applied to extract raw intensities. minfi (v. 1.52.1 [27]) was used for Illumina background correction to extract detection *p* values and information on biological sex, which was compared with the phenotypic information. The cutoff for detection *p* values was set to 1e^–16^. CpG sites were excluded if the call rate was below 98%, the bead count was lower than three, and if probes were cross-reactive or in close proximity to SNPs with a minor allele frequency larger than 1% in individuals from European ancestry [28, 29]. Exclusion criteria for samples were a call rate below 95% or a mismatch between reported and biological sex. No samples had to be excluded during quality control. After filtering, 709,315 CpG sites remained for analysis. Quantile normalization was applied, followed by calculating methylation values and subsequent log transformation into M values for analysis [30]. Cell type proportions were estimated using EpiDISH [31], using the IDOL-optimized reference data from Salas et al. (2018) [32]. Because of multicollinearity, all cell type proportions were included in the statistical models except for the neutrophil proportion.

### Statistical analysis

All statistical analyses were performed in the R statistical environment (version 4.4.2 [33]). To investigate whether DNA methylation at baseline was predictive of cortisol reactivity, robust linear models were used with the rlm function of the MASS package (version 7.1-63 [34]); see also Fig. 1 for the whole bioinformatics workflow). Cortisol reactivity, as indexed by AUCi, was predicted by M values of methylation, controlling for age, sex, self-reported smoking, cell type proportions, and five principal components (PC) of the positive control probes to control for batch effects and technical variation. Variance inflation analysis revealed that control probe PC3 was highly colinear with sex (r = 0.95), and we, therefore, excluded PC3 from the model (see also equation 1 in Fig. 1).

To investigate changes in DNA methylation levels after acute stress, mixed linear models were applied using the lmer function of lme4 (v.1.1-35.5 [35]), with M values as the independent variable, predicted by time, including subject as a random intercept, and controlling for age, sex, smoking, cell type proportions and the same principal components of the internal control probes as mentioned above. Singular results were excluded from further analyses. All results were corrected for multiple testing using the False Discovery Rate (FDR), and FDR values < 0.05 were considered epigenome-wide significant and included in downstream analyses. Additionally, we used bacon to further correct results. Results were annotated using the manufacturer’s manifest, based on hg19/GRCh37.

Differentially methylated regions (DMRs) were investigated using the dmrff algorithm (version 1.1.2 [36]), with a seed *p*-value of 0.001 and a window of 500 base pairs. DMR analysis was performed for all analyses with epigenome-wide significant findings. Results were visualized using the gwaRs package (version 0.3.0; [37]).

To identify biological mechanisms associated with the obtained findings, we annotated all CpG sites with a *p*-value < 0.001 to genes using the manufacturer’s manifest. After filtering, 688 genes from 768 CpG sites were tested for the AUCi model, and 1411 genes from 2125 CpG sites for the longitudinal model. Then, Gene Ontology (GO) overrepresentation analysis was performed using clusterProfiler (version 4.14.4 [38]), with a *p*-value cut-off of 0.05 and a q-value cutoff of 0.1. Semantic GO term similarity was assessed, and results were visualized using enrichplot (version 1.26.3).

The overlap of CpGs associated with cortisol AUCi with a previously published analysis by Houtepen et al. (2016) [20] was investigated for all nominally significant differentially methylated CpG sites.

## Results

### Sample description

After two participants were excluded because of missing cortisol information, data from 120 participants remained. As previously reported [23], the stress manipulation resulted in significant increases in both cortisol levels and perceived stress. The mean salivary cortisol profile and subjective stress levels are shown in Supplementary Fig. S1. The mean age of this final sample was 27.95 years, with a standard deviation of 7.44 years. Seventy-one participants (59%) were female, and 17 (14%) reported to be current smokers.

### EWAS of cortisol reactivity

**Fig. 2:**
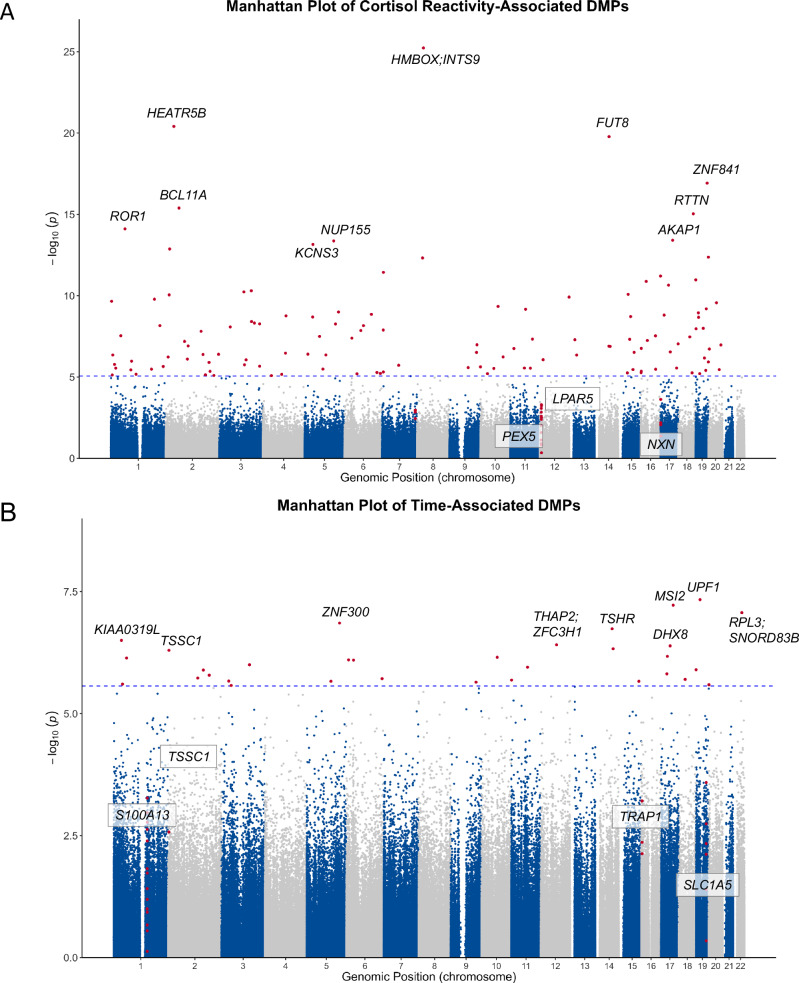
Manhattan plots of differential methylation associations. **A** Logarithmic *p*-values of CpG sites predicting cortisol reactivity (AUCi). **B** Logarithmic *p* values of CpG sites associated with time in the longitudinal analysis. DMP differentially methylated position, AUCi area under the curve with respect to cortisol increase. Red dots represent epigenome-wide significant CpG sites. The dotted line indicates epigenome-wide significance (FDR < 0.05). Genes annotated to DMPs are depicted in italics, and genes annotated to differentially methylated regions are additionally framed.

When predicting cortisol reactivity, as indexed by AUCi, with baseline DNA methylation levels, 120 epigenome-wide significant CpG sites emerged. The statistically strongest association was observed for cg07330094, annotated to the Homeobox containing 1 (*HMBOX1*) and Integrator complex subunit 9 (*INTS9*) genes, respectively (B = –3.18, SE = 0.30, *p* = 5.8*10^–26^, FDR = 4.11*10^–20^). All associations are depicted in Fig. 2A and Supplementary Table S1, and the corresponding QQ-plots for bacon corrected and uncorrected *p*-values can be found in Supplementary Fig. S2. The top 40 differentially methylated CpG sites associated with cortisol reactivity are shown in Table 1. Four DMRs were associated with cortisol reactivity: chr12:6745367-6745707 in the Lysophosphatidic Acid Receptor 5 (*LPAR5*) gene (z = –6.45, *p*_adj_ = 7.53*10^–5^), chr12:7341252-7341644 in the Peroxisomal Biogenesis Factor 5 (*PEX5)* gene (z = -5.46,* p*_adj_ = 0.034), chr17:707032-707228 in the Nucleoredoxin (*NXN*) gene (z = 5.45, *p*_adj_ = 0.036), and chr7:150872023-150872218 (z = –5.47, *p*_adj_ = 0.033), located in an intergenic region (see Supplementary Table S2).

**Table 1:** Top 40 differentially methylated CpG sites associated with cortisol reactivity, indexed by AUCi.

ProbeID	CHR	POS	Effect	SE	*P*	*N*	Gene	FDR
cg07330094	8	28747472	–3.184	0.302	5.805E–26	120	*HMBOX1;INTS9*	4.117E–20
cg18637383	2	37308026	–2.831	0.300	3.888E–21	120	*HEATR5B*	1.379E–15
cg22128147	14	65878037	–6.775	0.730	1.642E–20	120	*FUT8*	3.883E–15
cg11383279	19	52599232	–3.291	0.385	1.187E–17	120	*ZNF841*	2.104E–12
ch.2.1382002 F	2	60745480	1.784	0.219	4.038E–16	120	*BCL11A*	5.728E–11
cg18824571	18	67873340	3.256	0.405	9.105E–16	120	*RTTN*	1.076E–10
cg11837766	1	64239631	–3.636	0.468	7.716E–15	120	*ROR1*	7.818E–10
cg02501365	5	131832805	–3.296	0.436	4.260E–14	119		3.358E–09
cg18265938	17	55162298	–3.564	0.471	3.856E–14	120	*AKAP1*	3.358E–09
cg22433285	5	37371555	1.949	0.260	6.986E–14	119	*NUP155*	4.955E–09
cg09282497	2	18059200	–3.428	0.463	1.328E–13	120	*KCNS3*	8.565E–09
cg03142586	19	58220080	–3.199	0.441	4.227E–13	120	*ZNF154*	2.498E–08
cg22978087	8	24814126	–3.461	0.479	4.747E–13	120	*NEFL*	2.590E–08
cg14528254	7	5862839	–5.843	0.841	3.669E–12	120	*ZNF815*	1.859E–07
cg02174379	16	90085915	–9.833	1.430	6.127E–12	119	*DBNDD1*	2.897E–07
cg21067965	19	1067596	–3.636	0.535	1.046E–11	120	*HMHA1*	4.637E–07
cg17286200	16	25060966	–12.999	1.921	1.313E–11	120		5.480E–07
cg09463047	17	36104218	–3.539	0.529	2.240E–11	120	*HNF1B*	8.828E–07
cg00506629	3	145878177	2.586	0.393	4.940E–11	120	*PLOD2*	1.844E–06
cg00785783	3	112280577	1.912	0.292	5.756E–11	120	*ATG3;SLC35A5*	2.041E–06
cg19968840	15	45409319	1.824	0.281	8.254E–11	118	*DUOXA2*	2.788E–06
cg26010191	2	15701432	–3.094	0.477	8.830E–11	120	*NBAS*	2.847E–06
cg12075445	12	132906010	1.807	0.281	1.205E–10	120	*GALNT9*	3.716E–06
cg18847874	1	198126386	–7.319	1.145	1.630E–10	120	*NEK7*	4.818E–06
cg05254257	1	3371334	–3.076	0.485	2.181E–10	120	*ARHGEF16*	6.188E–06
cg24682316	20	35808040	–3.547	0.562	2.695E–10	120	*C20orf132;RPN2*	7.352E–06
cg22864549	10	80733765	–2.815	0.451	4.491E–10	120	*LOC283050*	1.180E–05
cg13402773	19	49140668	–3.027	0.490	6.359E–10	120	*SEC1;DBP*	1.611E–05
cg06223539	11	70517374	–6.924	1.122	6.736E–10	120	*SHANK2*	1.648E–05
cg23024136	5	153853154	–6.457	1.057	9.952E–10	120		2.353E–05
cg10362865	19	12721409	–1.965	0.323	1.119E–09	120	*ZNF791;ZNF490*	2.560E–05
ch.6.122375039 R	6	122333340	–3.002	0.496	1.388E–09	120		3.076E–05
cg23401912	17	16256974	3.482	0.577	1.543E–09	120	*CENPV*	3.317E–05
cg00911488	4	106068604	–2.819	0.468	1.704E–09	119	*TET2*	3.555E–05
cg13184270	15	56757290	–3.550	0.591	1.908E–09	120	*MNS1*	3.866E–05
cg22510662	5	36202853	–1.337	0.223	2.022E–09	119	*C5orf33*	3.984E–05
cg17750139	19	13842571	1.861	0.311	2.106E–09	120		4.037E–05
cg12836959	3	147098568	1.688	0.286	3.843E–09	120		7.173E–05
cg19459508	3	160282885	–2.860	0.488	4.768E–09	120	*KPNA4*	8.672E–05
cg13775832	3	184080450	–3.172	0.544	5.334E–09	120	*CLCN2;POLR2H*	9.420E–05

### Longitudinal DNA methylation change

We identified 32 CpG sites that were significantly differentially methylated after the stress test (see Table 2). After bacon correction, three CpG sites remained statistically significant. The strongest association was observed for cg02930615 (B = 0.11, SE = 0.02, *p* = 4.6*10^–8^, FDR = 0.016, FDR_bacon_ = 0.045) in the UPF1 RNA Helicase and ATPase (*UPF1*) gene. Differentially methylated CpG sites in known regulatory features were cg11192800 in the THAP Domain Containing 2 (*THAP2*) gene (B = –0.11, SE = 0.02, *p* = 4.55*10^–7^, FDR = 0.0299), cg09248944 in the CD302 Molecule (*CD302*) gene (B = -0.14, SE = 0.03, *p* = 1.43*10^–6^, FDR = 0.040), and cg13396682 in the Upstream Binding Protein 1 (*UBP1*) gene (B = 4.48, SE = 0.91, *p* = 2.11*10^–6^, FDR = 0.046). The QQ-plots for bacon corrected and uncorrected *p*-values can be found in Supplementary Fig. S3. Based on the summary statistics of this analysis, we identified four differentially methylated regions. The top three DMRs were chr1:153599487-153600156 in the S100 Calcium Binding Protein A13 (*S100A13*) gene (z = 8.25, p_adj_ = 9.92*10^–11^), chr16:3725478-3726052 in the TNF receptor associated protein 1 (*TRAP1*) gene (z = 5.80, p_adj_ = 4.02*10^–3^), and chr2:3325158-3325589 in the tumor suppressing subtransferable candidate 1 (*TSSC1*) gene (z = 5.68, p_adj_ = 8.04*10^–3^). Differentially Methylated Positions (DMPs) are listed in Supplementary Table S3 (*p* < 0.05), DMRs in Supplementary Table S4, and results are depicted in Fig. 2B and methylation levels of the DMPs significant after bacon correction can be found in Supplementary Fig. S4.

**Table 2:** Longitudinally differentially methylated CpG sites (before and after acute stress).

ProbeID	CHR	POS	Effect	SE	*P*	*N*	Gene	FDR
cg02930615	19	18966003	0.109	0.019	4.604E–08	120	*UPF1*	0.0169
cg17045539	17	55709884	0.083	0.015	5.983E–08	120	*MSI2*	0.0169
cg22041137	22	39709853	0.185	0.033	8.509E–08	120	*RPL3;SNORD83B*	0.0169
cg02343823	5	150284419	0.124	0.023	1.391E–07	120	*ZNF300*	0.0207
cg02168606	14	76583406	0.079	0.015	1.826E–07	120		0.0217
cg00027081	2	3325158	0.161	0.031	5.023E–07	120	*TSSC1*	0.0299
cg06026520	14	81597073	0.110	0.021	4.679E–07	120	*TSHR*	0.0299
cg11192800	12	72057255	–0.112	0.021	3.871E–07	120	*THAP2;ZFC3H1*	0.0299
cg11302666	17	41564564	0.139	0.026	4.082E–07	120	*DHX8*	0.0299
cg20083224	1	35928221	–0.098	0.018	3.142E–07	120	*KIAA0319L*	0.0299
cg10472021	1	59271497	0.112	0.022	7.233E–07	120	*LINC01135*	0.0317
cg13335769	6	33238465	0.076	0.015	7.983E–07	120	*VPS52;RPS18*	0.0317
cg14114133	6	10839408	–0.074	0.014	7.896E–07	120		0.0317
cg15881168	17	28916490	0.110	0.021	6.699E–07	120	*LRRC37BP1*	0.0317
cg24392133	10	71636063	0.065	0.013	6.981E–07	120	*COL13A1*	0.0317
cg22279328	3	128185869	0.221	0.043	9.942E–07	120	*DNAJB8;DNAJB8-AS1*	0.0370
cg15057214	11	74699344	0.110	0.022	1.116E–06	120	*NEU3*	0.0391
cg08162808	19	1008591	0.103	0.020	1.259E–06	120	*GRIN3B*	0.0400
cg09248944	2	160654499	–0.138	0.028	1.276E–06	120	*CD302*	0.0400
cg22640868	17	26661374	–0.062	0.013	1.528E–06	120	*TNFAIP1;IFT20*	0.0455
cg05014837	2	135059316	–0.126	0.025	1.858E–06	120	*MGAT5*	0.0461
cg10950033	6	163736276	–0.169	0.034	1.916E–06	120	*PACRG;LOC285796*	0.0461
cg11669049	11	1790443	0.105	0.021	2.048E–06	120		0.0461
cg13396682	3	33481969	4.479	0.911	2.146E–06	120	*UBP1*	0.0461
cg15860848	2	187409740	0.146	0.029	1.626E–06	120		0.0461
cg16976547	15	91433530	0.187	0.038	2.167E–06	120	*FES*	0.0461
cg18038361	18	29170604	0.093	0.019	1.982E–06	120	*TTR*	0.0461
cg19011122	5	110835117	0.148	0.030	2.163E–06	120	*STARD4*	0.0461
cg14485581	9	115912047	0.119	0.024	2.265E–06	120	*SLC31A2*	0.0465
cg03058862	1	40725250	0.134	0.028	2.478E–06	120	*ZMPSTE24*	0.0490
cg26617335	20	639881	–0.126	0.026	2.552E–06	120		0.0490
cg04604638	3	44688991	0.168	0.035	2.642E–06	120	*ZNF197;ZNF35*	0.0491

### Gene ontology overrepresentation analysis

Regarding the prediction of cortisol reactivity, the strongest overrepresentations were observed for the biological processes neurotransmitter transport (GO:0006836; *p* = 8.01*10^–5^, q = 0.12) and Wnt signaling (GO:0016055; *p* = 1.7*10^–4^, q = 0.12). However, none of the observed associations remained significant after multiple testing correction. There were no significant GO overrepresentations for molecular functions. At the same time, cellular components overrepresented in the AUCi prediction results were related to nuclear growth, actomyosin filament, and synaptic vesicles (see also Supplementary Table S5 and Supplementary Fig. S5).

**Fig. 3:**
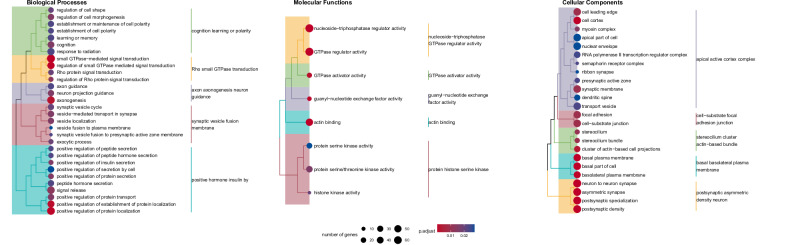
Overrepresented gene ontology (GO) terms for results of the longitudinal analysis. Colors represent semantically similar GO terms, circle size relates to the number of genes in GO terms, circle color indicates significance. P.adjust = adjusted *p*-value.

For longitudinal DNA methylation changes over the course of stress induction, Gene Ontology Overrepresentation analysis revealed a strong enrichment for biological processes related to hormone secretion, in particular insulin secretion (Fig. 3). In addition, we observed GO term overrepresentation implicating cognition and learning, Rho and small GTPase signal transduction, axonogenesis and neuron guidance, and synaptic vesicles. Molecular functions included GTPase activity, guanyl-nucleotide exchange factor activity, actin binding, and protein serine kinase activity. Cellular components included cortex and synapse-related terms, the stereocilium, the plasma membrane, and postsynaptic terms. All significant GO terms associated with time are listed in Supplementary Table S6.

### Comparison of AUCi prediction results with Houtepen et al. (2016)

Results of the AUCi prediction were cross-referenced with the results by Houtepen et al. (2016) [20]. Of the 22,425 nominally significant CpG sites from Houtepen et al., 601 were also differentially methylated at nominal significance in the present study. At the same time, only 288 (47.9%) exhibited convergence in the direction of effect (Supplementary Fig. S6), and the top association from Houtepen et al. with cg27512205 did not replicate in the present study (B = 13.85, *p* = 0.42). Summary statistics for the 601 CpG sites found in both studies are listed in Supplementary Table S7.

## Discussion

The present study identified 120 CpG sites prospectively associated with cortisol stress reactivity following an acute laboratory stressor. Moreover, longitudinal DNA methylation changes from before to after acute stress induction were observed at 32 CpG sites and four DMRs, suggesting temporal dynamics of DNA methylation with changes at a genome-wide level in response to acute stress.

The top hit was annotated to the Integrator Complex Subunit 9 (*INTS9*) and the Homeobox Containing 1 (*HMBOX1*) genes when investigating associations between cortisol reactivity and baseline DNA methylation levels. INTS9 is a subunit of the Integrator complex, which binds to RNA polymerase II [39]. HMBOX1 plays a critical role in telomere elongation and enables TERT chromatin binding [40]. In addition, we observed differential methylation in several genes representing chromatin and methylation modifiers, such as the Histone Deacetylase 4 (*HDAC4*) and Tet-Methylcytosin-Dioxygenase 2 (*TET2*) genes. This might suggest cortisol reactivity to be dependent on chromatin states. On a cellular level, chromatin modifiers have been identified as key stress response regulators [41], and histone modifications were implicated in animal models of stress [42]. In addition, many of the most strongly associated CpG sites were located in transcription factor genes, including Zinc Finger Proteins, such as *ZNF841*, *ZNF815*, and *ZNF154*. Therefore, the present results align with the human stress response involving both chromatin remodeling and transcription factor activity [43]. Furthermore, among the top results, a CpG site in the SH3 And Multiple Ankyrin Repeat Domains 2 (*SHANK2*), a key risk gene for autism spectrum disorders (ASD), was identified. Mutations in *SHANK2* have been discussed as monogenetic causes for ASD [44] and are used to model ASD in mice [45]. Mechanistically, SHANK mutations are associated with altered neurotransmission and synaptic circuitry defects [44]. Given its role in neurotransmission and synaptic function, differential methylation in *SHANK2* may provide insights into the molecular mechanisms of cortisol reactivity in response to acute stress.

When comparing the present results to those by Houtepen et al. [20], there was limited overlap between the two studies. This could be due to differences in sample composition, as the present study included healthy volunteers in contrast to individuals with early trauma and, in part, clinically relevant depressive symptoms in Houtepen et al. (2016). Trauma exposure has consistently been linked to a blunted cortisol response (e.g., [46, 47]), as has depression [48], thus reducing the comparability between the two cohorts. On a methodological level, differences in study design or analysis pipelines might also contribute to the lack of overlap between the studies. Therefore, sufficiently powered sample sizes and meta-analyses of epigenome-wide association studies of the acute stress response are warranted to identify robust signals.

Among the top longitudinal findings, two genes emerged related to thyroid functioning: *TSHR* coding for the thyroid stimulating hormone (TSH)-receptor and *TTR* transthyretin. This is in line with previous research observing altered levels of TSH after acute stress induction by the Trier Social Stress Test (TSST) [49]. Similar results have been observed in rats that underwent acute and chronic stress paradigms: here, TSH levels were found to be increased in response to acute stress but blunted in chronic stress [50]. Interestingly, altered thyroid function has robustly been associated in subgroups of patients with depression [51] and anxiety disorders [52]. A thyroid autoimmune subtype could also play a role in schizophreniform and affective disorders, as a recent study investigating 530 patients with schizophreniform and affective disorders observed thyroid antibodies in about 17% of patients [53]. Similarly, in a cohort of 100 patients with unipolar depression, 17% of patients exhibited thyroid antibodies [54]. Next to autoimmune thyroiditis, symptoms of hypothyroidism, such as fatigue, depressive mood, and cognitive dysfunction, and hyperthyroidism, including nervousness, anxiety, and irritability, show clinical similarities to symptoms of depression and anxiety, respectively [55]. Thus, the present findings support the role of thyroid-associated factors in acute stress that might influence the development of depression and anxiety disorders.

In the GO term analysis of genes implicated in the longitudinal analysis, GO terms related to hormone secretion, particularly insulin secretion, were overrepresented. Acute stress has been observed to induce higher plasma insulin levels in a rodent model, especially in animals with early-life stress [56]. Additionally, male patients with stress-related exhaustion exhibited increased fasting serum insulin concentrations, providing additional evidence for increased insulin secretion due to stress [57]. Therefore, the observed pathway enrichment for insulin secretion may indicate an activation of stress-associated mechanisms.

While the implications of acute stress–related epigenetic changes for the development of stress-related mental disorders remain to be clarified, such changes may also be informative for understanding mechanisms of therapeutic action. Acute stress paradigms may also provide a useful model for investigating extinction-related mechanisms relevant for exposure-based treatments, as supported by findings of dynamic DNA methylation changes during fear exposure (cf. [58]).

Several limitations apply to the present study. First, while DNA methylation has potential as a biomarker, we can only speculate about the functional consequences of the present findings, as DNA methylation has a complex relationship with gene and protein expression [59]. In addition, statistical power remains a limitation, considering the sample size compared with the number of tests performed. Future studies with larger sample sizes are needed to provide evidence from multiple “omics”-levels and to perform functional validation experiments. Second, the measured stress-induced DNA methylation changes at significant CpG sites were of low magnitude, potentially indicating a fine-tuned biological response rather than broad epigenetic remodeling, and their potential biological relevance warrants further investigation. Furthermore, it is unclear whether the observed changes are persistent, and follow-up studies with longer observation periods are needed to investigate the stability of DNA methylation patterns at later time points post-stress. Nevertheless, it is noteworthy that any differences could be detected in peripheral tissue within such a short observation period. Blood is particularly suitable as a peripheral marker for stress-related processes in the brain as DNA methylation patterns correlate with those in the brain [60] and immune cells can respond quickly to stress signals. Stress hormones such as cortisol and catecholamines can not only alter cell composition but also change gene expression of immune cells in blood rapidly [61, 62]. Moreover, a recently published study on acute stress showed that DNA methylation in saliva, containing immune cells, changes after acute stress [63]. A study on the stability of DNA methylation during acute stress in healthy individuals and individuals with early life adversity also showed that acute stress can cause changes in DNA methylation, particularly in CpGs used to calculate cell composition, making it difficult to rule out false DNA methylation changes due to changes in cell proportion. To avoid this confounding factor, future studies should not determine blood cell composition based solely on epigenetic algorithms, but instead determine cell proportions directly [64].

The present study suggests that DNA methylation predicts cortisol reactivity and that acute stress may induce rapid changes in methylation levels within one hour. Our results highlight genes involved in thyroid function, hormone secretion, chromatin remodeling and transcriptional regulation to play a role in the stress response. Although these results provide valuable insights into the interplay between DNA methylation and stress reactivity, further studies are needed to confirm these findings, examine their long-term stability, and investigate their functional implications and their adaptive or maladaptive role regarding stress-related disorders. Future research in larger sample sizes and employing multi-omics approaches are essential to better elucidate the complexity of epigenetic regulation of the stress response.

## Supplementary information

Supplementary Figures

Supplementary Tables

## Data Availability

The data that support the findings of this study are available from the corresponding author on reasonable request,
