# Supplementary material for: Acute stress induces changes in epigenome-wide DNA methylation
Source: Neuropsychopharmacology. 2025 Dec 12;51(4):732–40. doi: 10.1038/s41386-025-02289-8 (PMC12932828; doi:10.1038/s41386-025-02289-8)
Supplement: Supplementary file 1 — Supplementary Figures [file 41386_2025_2289_MOESM1_ESM.docx]

**Supplementary Material**

A)

B)


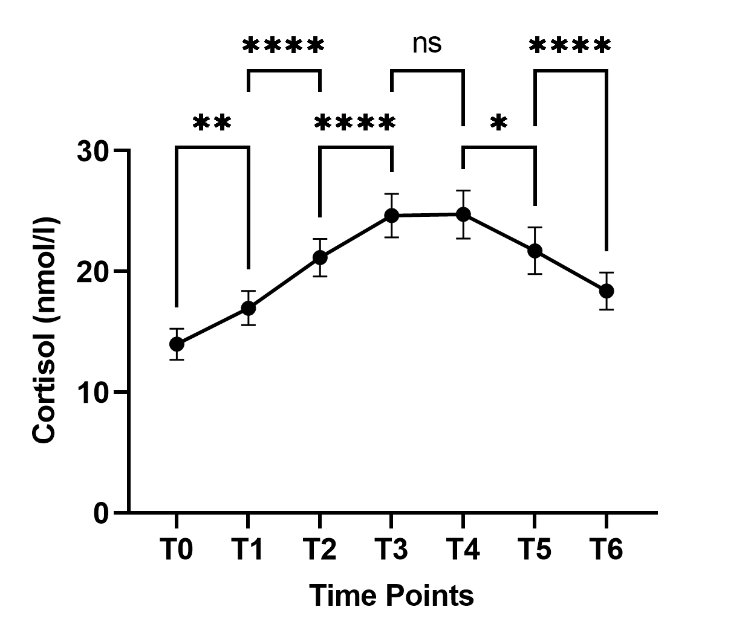

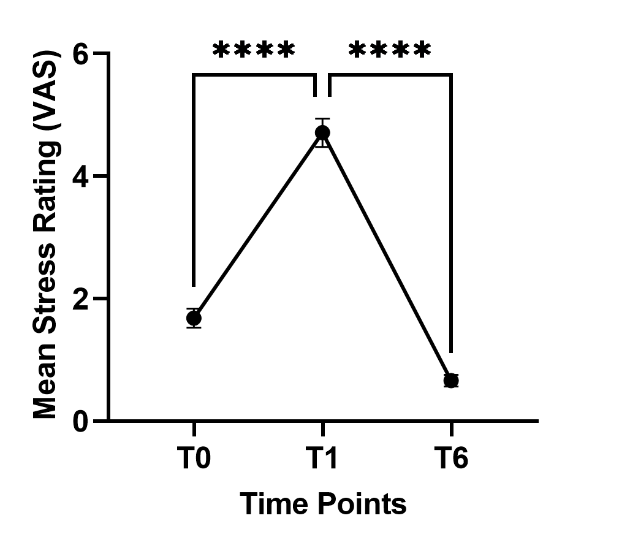


Supplementary Figure S1. A) Mean salivary cortisol concentrations (± SEM) before and after the stress test across six measurement time points. B) Mean subjective stress ratings (± SEM) before (T0), directly after (T1), and 45 minutes after (T6) the stress test. The grey areas between T0 and T1 denote the duration of the stress test. Asterisks indicate a statistically significant change in consecutive cortisol levels and stress ratings, respectively, **p* < 0.05, ***p* < 0.01, ***p* < 0.001, *****p* < 0.0001 (Tukey‘s multiple comparisons test). VAS: Visual Analogue Scale. SEM: Standard Error of the Mean.


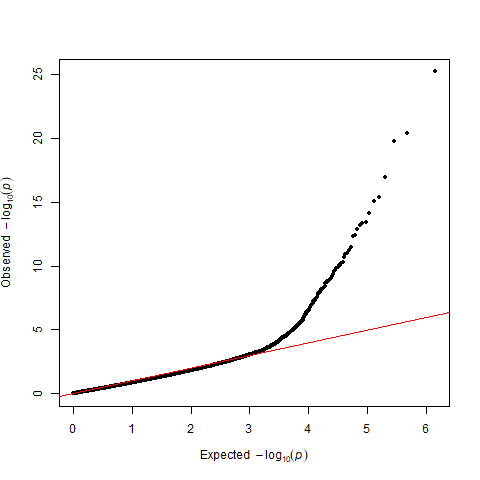

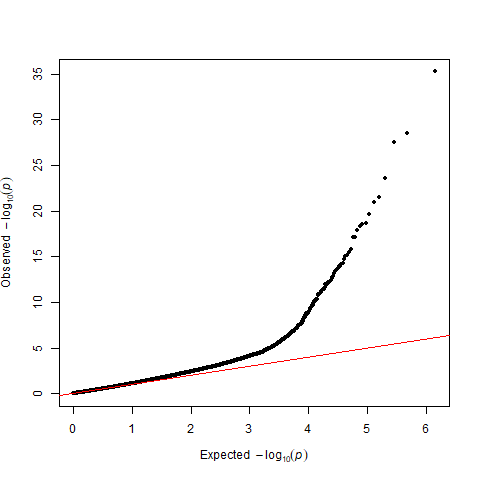


A)

B)

Supplementary Figure S2. A) QQ-plot for AUCi model, lambda=0.73. B) QQ-plot for bacon-corrected AUCi model, lambda = 1.03


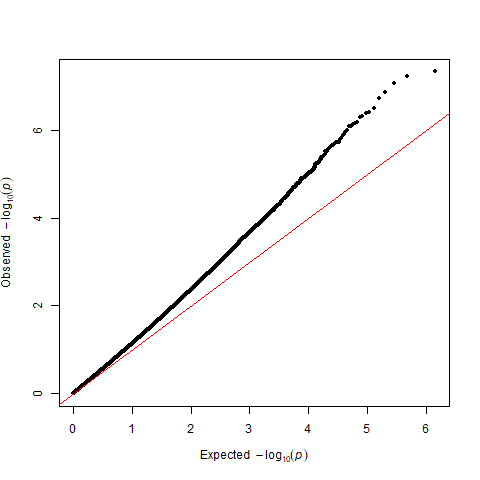

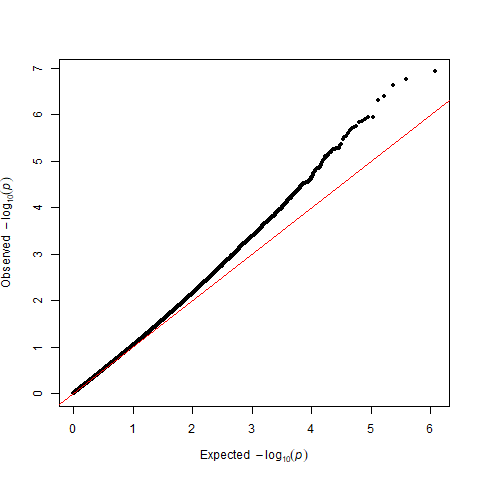


B)

A)

Supplementary Figure S3. QQ-plot for time, lambda= 1.18. B) QQ-plot for bacon-corrected time model, lambda = 1.03


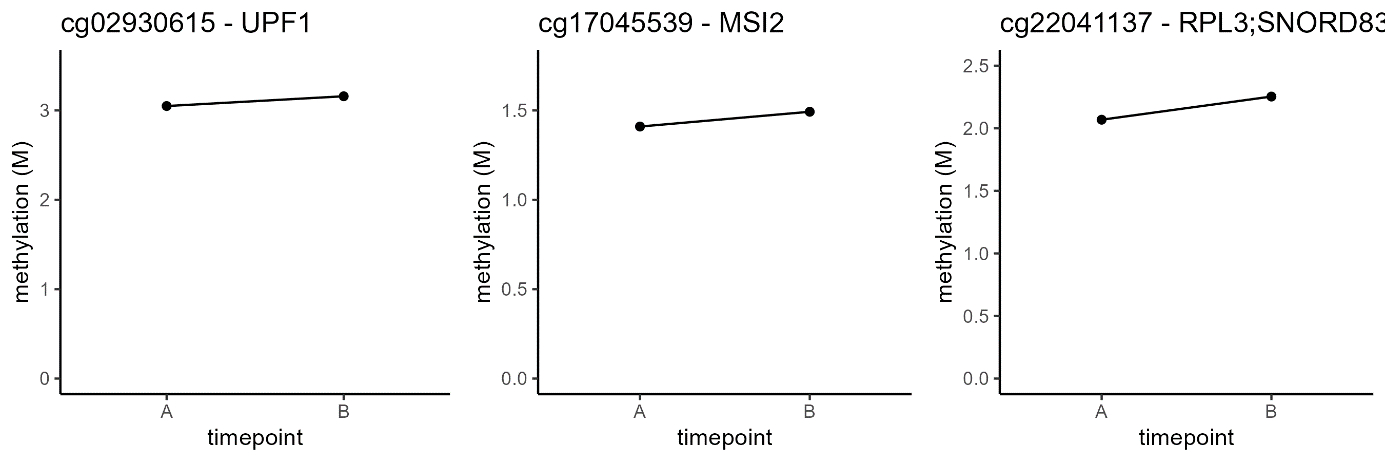


Supplementary Figure S4. Methylation levels (M values) based on estimated marginal means from the longitudinal model for the three CpG sites significant after bacon correction.


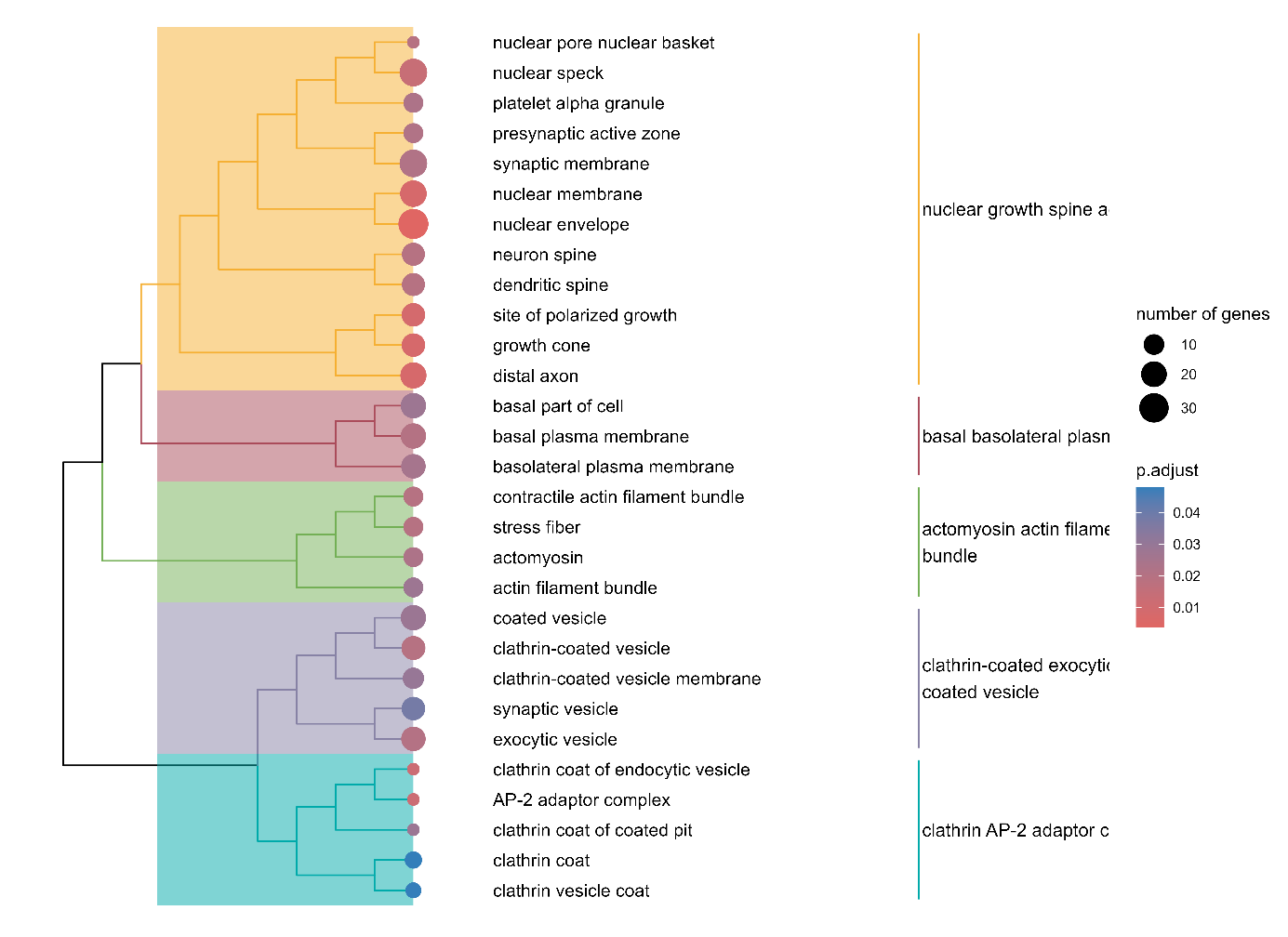


Supplementary Figure S5. Gene Ontology Overrepresentation: Cellular Components


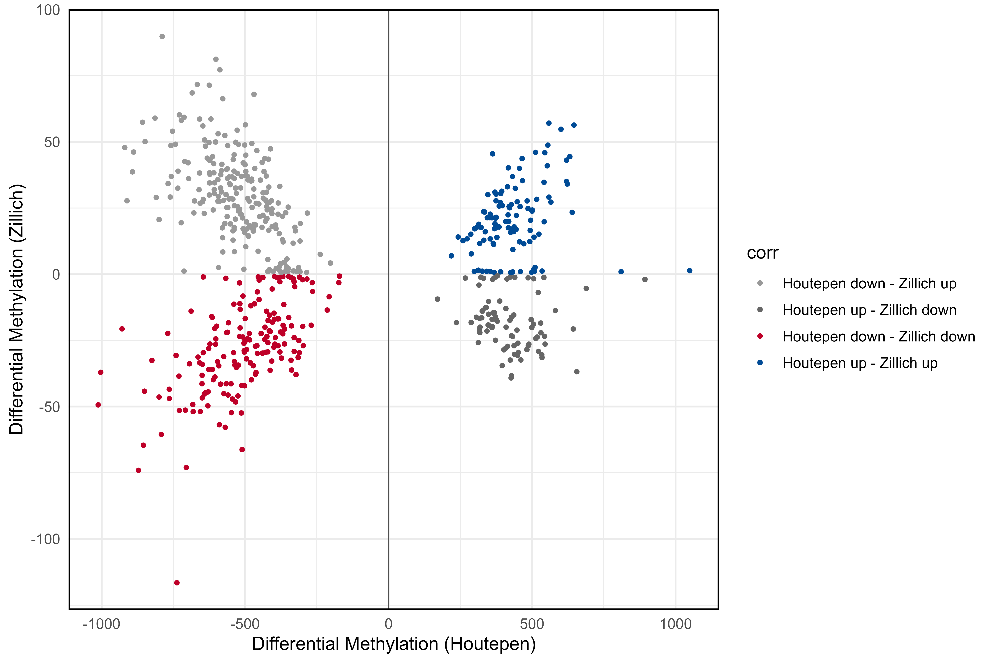


Supplementary Figure S6. Direction of effect of overlapping genes between Houtepen et al. (2016) and the present study.
